# Amyloid Beta_1-40_-Induced Astrogliosis and the Effect of Genistein Treatment in Rat: A Three-Dimensional Confocal Morphometric and Proteomic Study

**DOI:** 10.1371/journal.pone.0076526

**Published:** 2013-10-09

**Authors:** Maryam Bagheri, Arjang Rezakhani, Sofie Nyström, Maria V. Turkina, Mehrdad Roghani, Per Hammarström, Simin Mohseni

**Affiliations:** 1 Department of Physiology, Faculty of Medicine, Ilam University of Medical Sciences, Ilam, Iran; 2 Department of Clinical and Experimental Medicine, Faculty of Health Sciences, Linköping University, Linköping, Sweden; 3 IFM-Department of Chemistry, Linköping University, Linköping, Sweden; 4 Department of Physiology, Neurophysiology Research Group, Shahed University, Tehran, Iran; Massachusetts General Hospital/Harvard Medical School, United States of America

## Abstract

Astrocytes are highly involved in regulation and homeostasis of the extracellular environment in the healthy brain. In pathological conditions, these cells play a major role in the inflammatory response seen in CNS tissues, which is called reactive astrogliosis and includes hypertrophy and proliferation of astrocytes. Here, we performed 3D confocal microscopy to evaluate the morphological response of reactive astrocytes positive for glial fibrillary acidic protein (GFAP) in rats, to the presence of Aβ_1–40_ in the rat brain before and after treatment with genistein. In 50 astrocytes per animal, we measured the volume and surface area for the nucleus, cell body, the entire cell, the tissue covered by single astrocytes and quantified the number and length of branches, the density of the astrocytes and the intensity of GFAP immunoreactivity. Injecting Aβ_1–40_ into the brain of rats caused astrogliosis indicated by increased values for all measured parameters. Mass spectrometric analysis of hippocampal tissue in Aβ_1–40_-injected brain showed decreased amounts of tubulins, enolases and myelin basic protein, and increased amounts of dihydropyrimidinase-related protein 2. In Aβ_1–40_-injected rats pretreated with genistein, GFAP intensity was decreased to the sham-operated group level, and Aβ_1–40_-induced astrogliosis was significantly ameliorated.

## Introduction

Astrocytes are highly involved in the regulation of extracellular ion and neurotransmitter homeostasis in the healthy brain [1-4] and failure of astrocyte-dependent homeostasis leads to imbalance in neurotransmission in a wide range of diseases [5]. Astrocytes also play a pivotal role in the modulation of synaptic plasticity that is important for mechanisms of cognition, learning, and memory [6,7]. These glia cells respond to harmful stimuli by changing their molecular, cellular, and functional properties. This response is known as reactive astrogliosis and is manifested as hypertrophy, proliferation, and functional remodeling [8]. In Alzheimer’s disease (AD), activation of astrocytes is initiated by pro-inflammatory factors and excessive nitrosative and oxidative stress [9]. Sofroniew et al. [10] postulate that reactive astrocytes protect the brain from insults by isolating the damaged area, reconstructing the blood-brain barrier, and rearranging the tissue structure. On the other hand, Garwood and colleagues [11] recently observed that Aβ-induced neuronal death was accelerated by the presence of astrocytes in primary culture, and this neuronal loss was reduced when astrocyte activation was inhibited by treatment with an anti-inflammatory drug. Such drugs have been used in patients as a therapeutic approach to delay the progression of AD, but, unfortunately, they have not achieved the desired effects. For example, Jaturapatporn and coworkers in 2012 noted that a non-steroidal anti-inflammatory drug (NSAID) failed to influence the progression of cognitive deterioration [12]. Overall, knowledge regarding the role of astrogliosis in AD is limited. 

Genistein is an isoflavone that is found in a number of plants and has been shown to have anti-oxidant and anti-inflammatory properties. This compound can decrease the level of inflammatory cytokines and inhibit the activity of nuclear factor-[kappa]B (NF_K_B) [13]. Recent studies of rats have demonstrated that genistein ameliorates both memory impairment [14] and Aβ_1–40_-induced neuronal death [15]. The effect of this compound on astrogliosis, however, is unknown. In the current investigation, we evaluated the morphological response of astrocytes to the presence of Aβ_1–40_ in the brain before and after treatment with genistein. In short, we used 3D confocal microscopy images to measure 12 different parameters, which revealed signs of hypertrophy in astrocytes exposed to Aβ_1–40_. In addition, the protein composition of the Aβ_1–40_ inoculated tissue was analyzed by mass spectrometry.

## Materials and Methods

### Ethics statement

This study was carried out in accordance with the policies set forth in the Guide for the Care and Use of Laboratory Animals (NIH), approved by Ethics Committee of Tehran University of Medical Sciences (Tehran, Iran), and was according to stipulated guidelines available online at http://vcr.tums.ac.ir/word_files/animal research.doc (In Persian). All surgery was performed under anesthesia (see below), and all efforts were made to minimize suffering.

### Animals

Eighteen adult male Wistar rats (age 5 months ± 1 week; weight 250–300 g) were randomly assigned to four groups, which, respectively, were subjected to the following: sham operation (n = 4); injection of Aβ_1–40_ (n = 5); genistein pretreatment and subsequent Aβ_1–40_injection (n = 5); Cremophor EL pretreatment and subsequent Aβ_1–40_ injection (n = 4). Cremophor EL (0.5 ml) was used as a vehicle for genistein (10 mg/kg), and both agents were administered by gavage. In addition, three healthy and three Aβ_1–40_-injected rats were used for proteomic analysis by mass spectrometry.

To determine the conformational forms of the Aβ_1–40_ used in our study, a sample of the Aβ_1–40_ solution was analyzed using the Thioflavin T fluorescence assay. The results showed that the vast majority of the injected Aβ_1–40_ was in non-fibrillar form. The ThTassay showed relative fluorescence units at 480 nm (RFU) which was approximately 2% of the reference RFU for fully mature Aβ fibrils. 

### Surgery

Rats were anesthetized by intraperitoneal injection of ketamine (100 mg/kg) and xylazine (10 mg/kg) and immobilized in a stereotaxic instrument (Stoelting, IL, USA). The animals were then given 4 µl of normal saline (sham-operated group) or Aβ_1–40_ (2 nM; three experimental groups) bilaterally in the hippocampus at -3.5 mm posterior to bregma, ± 2 mm lateral to midline, and -2.8 mm below dura, according to the atlas of the rat brain [16]. Injections were performed over 4 min (1 µl/min) in each hemisphere using a Hamilton syringe with a 26S gauge needle. The needle was left in place for an additional 5 min and thereafter slowly retracted. 

### Specimen preparation for light microscopy

 Three weeks after the surgery, the animals were anesthetized with ketamine (150 mg/kg) and perfused with paraformaldehyde (4%) in 0.1 M PBS (pH 7.4). The brain of each rat was post-fixed and embedded in paraffin, and every third coronal sections (20 µm) of the right hippocampus were prepared to allow detection of GFAP by immunohistochemical staining. The sections used for morphometric analysis were taken between -3.3 and -4.5 posterior to bregma [16]. 

### Immunohistochemistry

For each animal, two separate hippocampal sections were deparaffinized and rehydrated, and then incubated with PBS containing normal serum (3.5%), triton X100 (0.25%), and bovine serum albumin (BSA, 0.25%) for 20 minutes. Thereafter, the sections were incubated for 5–10 min with serum-free protein block solution (Dako, Glostrup, Denmark) and subsequently overnight at 4° C with polyclonal rabbit antibodies against (GFAP; Dako, Glostrup, Denmark) diluted in PBS (1:1500) containing normal serum, triton X100, and BSA as described above. After washing in PBS, the sections were incubated with alkaline phosphatase-conjugated swine anti-rabbit IgG antibodies (1:100; Dako, Glostrup, Denmark) at room temperature for one hour. The sections were then washed in PBS and incubated for 15 min with Liquid Permanent Red Chromogen diluted in Liquid Permanent Red Substrate Buffer (Dako,Glostrup, Denmark). Finally, the nuclei were stained with 4'-6-diamidino-2-phenylindole (DAPI; diluted 1:500 in PBS) and mounted. Sections used as negative controls were run parallel with other sections but were omitted from primary antibodies i.e. GFAP antibodies. 

### Morphometric analysis of astrocytes

A confocal microscope (Zeiss LSM 700) was used to acquire images for morphometric analysis. In this assessment, we used two sections from each animal and captured 3D images (z-stack) of fifty astrocytes/animal. The cells that were photographed had a clearly visible cell body, a DAPI-stained nucleus, and no overlapping branches; these cells were located in an area between the hippocampal fissure and stratum granulare in the medial blade of the dentate gyrus (DGmb) in hippocampal formation. For the morphometric analysis, 3D images of 900 astrocytes from all animals were created from 14,000 consecutive 2D images included in the study taken at a uniform interval of 1.01µm. The X, Y, and Z properties of the images were 0.132, 0.132, and 1 µm/pixel, respectively. 

The length of branches was determined by using Easy Image Analysis^®^ 2000 software (Tekno Optic, Stockholm, Sweden) to manually draw individual branches. Volocity 5.5 (Perkin Elmer Inc., Massachusetts, USA) was used to measure the following for each astrocyte: the surface area and volume of the DAPI-stained nucleus, the cell body, and the entire cell (i.e., the cell body and branches), and the area and volume of the tissue covered by the astrocyte (designated the astrocyte territory). We performed a pilot study for the measurements and observed that the software recognized two cells that were very close to each other as one cell. Therefore, the boarder of the cells were manually determined on coded slides which was very time consuming but reliable. Delimitation of the astrocyte territory was achieved by drawing a line between the tips of the branches. The Perkin Elmer online support kindly assisted us and did a few measurements, and also calculated the surface area and volume of few cells by using the diameter of the cell to control if we had determined the measurements correctly.

The number of the primary- and the total number of branches was counted in 50 astrocytes/animal. Furthermore, the number of astrocyte cell body was counted in a well-defined area (117.000 µm^2^) including DGlb, DGmb and CA3 by using 2D pictures (x100). For this purpose, we counted the astrocytes exhibiting a clear cell body and a well-defined DAPI-stained nucleus and calculated the mean density of astrocyte cell body.

### Fluorescence intensity

In two separate hippocampal sections from each animal, we measured the intensity of GFAP^+^ immunoreactivity (arbitrary units) in an area of 0.7 mm^2^ located between stratum moleculare and the DGmb. 

### Preparation of brain homogenates for gel electrophoresis

In order to evaluate the biochemical changes caused by injection of Aβ_1–40_ peptide in the hippocampus of the rat, three healthy and three Aβ_1–40_-injected rats were deeply anesthetized (ketamine, 150 mg/kg) and decapitated by guillotine apparatus three weeks after the surgery. The brains were removed in less than 5 minutes, frozen instantly in liquid nitrogen and stored at -70 °C until used. At the time for analysis, the hippocampi were isolated, weighed and homogenized. The brain material was pestled and two equivalents of 0.32 M sucrose in PBS were added. Soluble and insoluble material was separated by centrifugation 8000g, 4° C, 15 min in table top centrifuge. The supernatant (soluble fractions) was removed, and the pelleted material (insoluble fraction) was resuspended in 5 M guanidinium thiocyanate, GdnSCN, using a volume equivalent to the removed supernatant. The GdnSCN was removed by dialysis back to sucrose-PBS buffer. 

### Capillary electrophoresis

Bioanalyzer 4200 (Agilent biotechnology) equipped with Protein 80 chip was used to analyze the homogenates of frozen tissue. The prepared soluble fractions of brain homogenates from right and left hippocampi were diluted 1:1 in the sucrose-PBS buffer. Electrophoresis samples were prepared and run according to manufacturer’s instructions. 

### SDS-PAGE and proteomic analysis

Based on the results from capillary electrophoresis, representative samples from control group and Aβ-injected group were selected for SDS-PAGE and subsequent proteomic analysis. The samples were fractionated in soluble and insoluble material. SDS-PAGE was run to perform in-gel tryptic digestion and mass finger printing. Criterion TQX 4-20% gel (Biorad, CA, USA) was used and electrophoretic separation was performed at 100 V for 1.5 h. After electrophoresis, gels were stained with Biosafe coomassie (Biorad, CA, USA). For in-gel digestion, the selected protein bands that showed obvious differences in protein amount between samples from healthy and Aβ_1–40_-injected tissues were excised and digested by trypsin according to Shevchenko et al. [17]. Obtained peptide mixtures were analyzed by LC-MS/MS. In short, the proteins were digested with trypsin. The resulting peptides were extracted from the gel with trifluoroacetic acid, dried, and stored at -20 °C until needed. The obtained peptide mixtures were analyzed by LC-MS/MS, using nano-flow HPLC system (EASY-nLC from Bruker Daltonics, Bremen, Germany) on a 20 mm x 100 μm (particle size 5 μm) C18 pre-column followed by a 100 mm x 75 μm C18 column (particle size 5 μm) at a flow rate 300 nL/min, using a linear gradient constructed from 0.1% formic acid (solvent A) to 0.1% formic acid in 100% acetonitrile (solvent B): 0–100% B for 60 min. Data were acquired by on-line electrospray ionization ion trap “HCT ultra PTM Discovery System” (Bruker Daltonics, Bremen, Germany) using collision-induced dissociation mode. Peak lists were created from the raw data using Bruker Daltonics Data Analysis 3.4 (Bruker Daltonics, Bremen, Germany) and the resulting MGF files were used to search for *Rattus* proteins in NCBI on the Mascot server (www.matrixscience.com). The search parameters allowed mass errors up to 0.8 Da for MS data, and up to 0.8 Da for MS/MS data. The charge states of the peptides were varied; one missed cleavage sites were permitted. Cysteine carbamidomethylation was selected as a fixed modification. N-terminal protein acetylation and methionine oxidation were selected as variable modifications. For identification of peptides we used the following criteria: the peptide MASCOT score was above 30, the significance threshold was set at 0.05 and redundant identifications were excluded using the bold red function. The above mentioned experiments were repeated 4 times for the capillary electrophoresis and 3 times with SDS-PAGE using in total 10 independent samples from both right and left hemispheres.

### Statistical analysis

All results were expressed as mean ± SEM, and GraphPad Prism 5 (GraphPad Software Inc., CA, USA) and SigmaStat^®^ 3.5 were used to assess the statistical differences. Parametric one-way ANOVA followed by Tukey’s post hoc test were used to compare the data between groups. In all analyses, a difference at *P* < 0.05 was regarded as significant. 

## Results

### The pattern of GFAP-positive astrocytes

Sections of the cerebral cortex were found to contain a large number of GFAP-positive (GFAP^+^) astrocytes around the site where the syringe needle had been inserted. However, the slides that were incubated without primary antibodies and served as negative controls showed no sign of immunoreactivity. These observations were made in sections taken from rats in all four groups, and thus they are not discussed further below.

**Figure 1:**
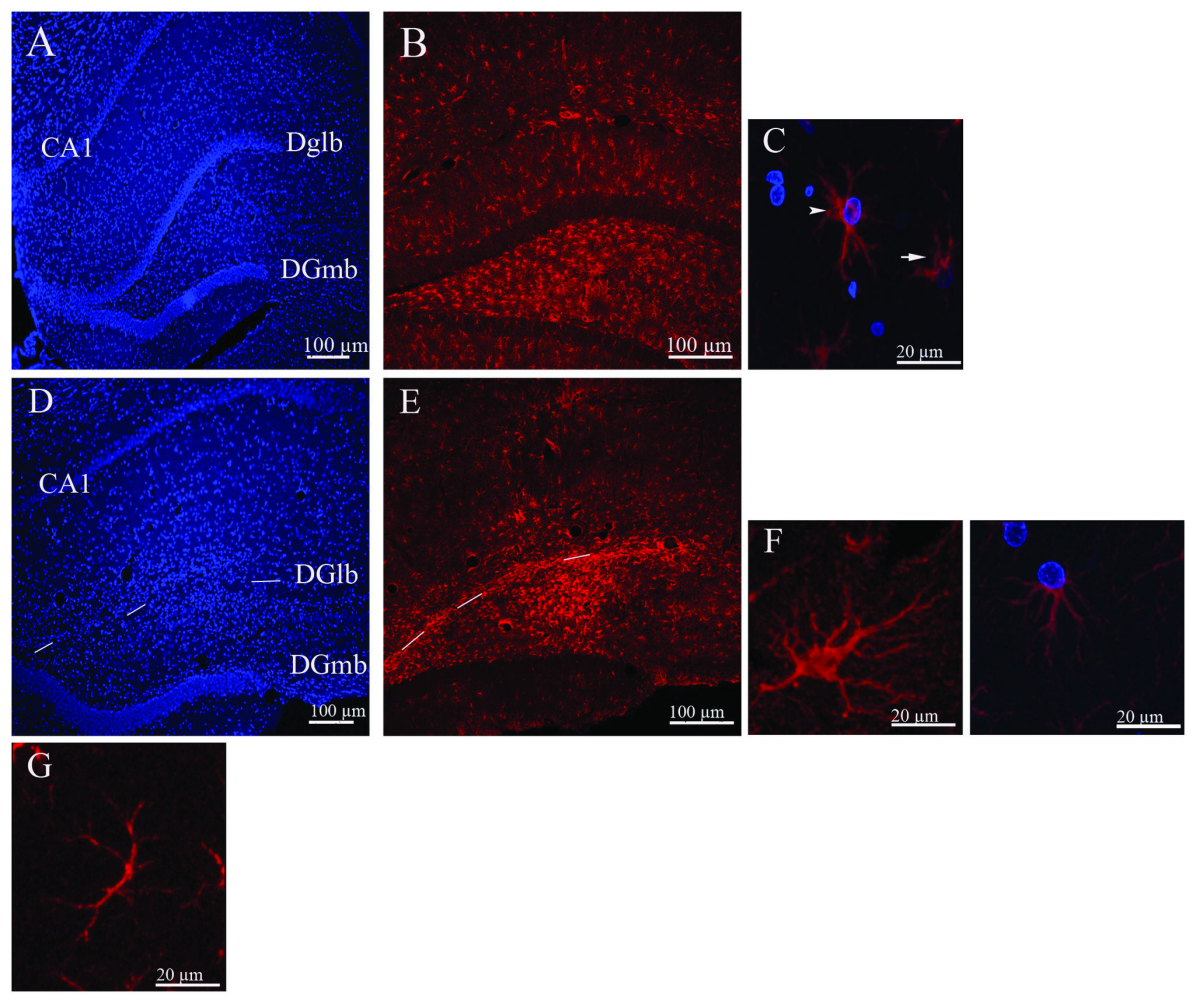
Confocal images of hippocampal sections obtained from rats subjected to sham operation (A–C) or injection of Aβ_1–40_ (D–F) in the hippocampus. **A**: DAPI-stained section showing normal architecture of the hippocampus. **B**:Image illustrating the pattern of GFAP immunoreactivity. **C**: Astrocyte branches were distributed either symmetrically (arrowhead) or asymmetrically (arrow) around the cell. **D**: DAPI-stained section showing abnormal architecture of the hippocampus; note the absence of the DGlb. **E**: GFAP immunoreactivity. **F**: individual astrocytes were either stellate in shape (**F-left**) or asymmetric with branches directed towards one side of the cell (**F-right**). **G**: An astrocyte lacking a distinct cell body, as observed in Aβ–genistein- and Aβ–Cremophor-EL-treated rats. Aβ_1–40_ (2 nM) was injected into the hippocampus. Abbreviations: DGlb, lateral blade of the dentate gyrus; DGmb, medial blade of the dentate gyrus; CA1, cornuammonis area 1. (**A, B, D, E**: 100 µm), (**C, F, G**: 20 µm).

*In The sham-operated group* (*n = 4*), the architecture of the hippocampus appeared normal in the DAPI-stained sections (Figure 1A). The GFAP^+^ astrocytes occurred throughout the cerebral cortex and hippocampus. In the cortex, they were small and sparsely distributed, and exhibited stellate morphology with multiple short branches. The occurrence of GFAP^+^ astrocytes increased from layer 1 towards layer 6 of the cortex and was most intense in corpus callosum and the polymorphic layer of the hippocampus (Figure 1B). In the hippocampus, the cornuammonis 1 (CA1) subfield contained few GFAP^+^ astrocytes with long branches, and the cornuammonis 2 (CA2) subfield displayed a dense network of small astrocytes with overlapping short branches. Both the DGmb and the lateral blade of the dentate gyrus (DGlb) showed weak GFAP immunoreactivity (Figure 1B). In the area that was in focus in our morphometric analysis, the branches of individual astrocytes appeared in either of two ways (Figure 1C): distributed symmetrically around the cell and thereby creating a stellate shape resembeling protoplasmic astrocytes, or asymmetrically arborized and pointed towards one side of the cell, with the nucleus located laterally in the cell body resembling fibrous astrocytes.

In *the Aβ*_*1–40*_ *-injected group* (*n = 5*), the DAPI-stained sections showed signs of neuronal loss in the DGlb of all the animals (Figure 1D). As observed in the serial sections, this cell loss affected the whole hippocampus. The cerebral cortex of these animals contained only a few small stellate-shaped GFAP^+^ astrocytes exhibiting short branches. The corpus callosum contained a dense network of GFAP^+^ cells in two of the rats but only few such cells were observed in the other three animals. The hippocampus from the CA1 subfield to the polymorph layer of the dentate gyrus displayed extensive signs of GFAP immunoreactivity, particularly in the area of the DGlb that exhibited severe loss of neurons (Figure 1E). The CA2 contained only a few GFAP^+^ astrocytes, and the DGmb showed negative GFAP immunoreactivity (Figure 1E). Overall, astrocytes in the hippocampus of the rats in this group had multiple long thin or thick branches. Also, most of the astrocytes were stellate in shape (Figure 1F left), and in some the nucleus was located laterally, and the branches were directed toward one side of the cell (Figure 1F right). In most astrocytes, the GFAP^+^ immunoreactivity had a punctuate appearance along the length of the branches. 

*In The group treated with Aβ*_*1–40*_ *and genistein* (*n = 5*), the DAPI-stained sections showed signs of neuronal loss in the DGlb in three of the animals and appeared normal in the other two. In the cortex, the occurrence of GFAP^+^ astrocytes increased from layer 1 towards layer 6 in three of the rats, whereas such immunoreactivity was absent in the other animals. Corpus callosum and the CA1 subfield of the hippocampus contained few GFAP^+^ astrocytes in this group. The immunoreactivity was extremely high in the DGlb and the polymorphic layer of the hippocampus in the animals that exhibited neuronal degeneration, whereas it was weak in those that had a normal DGlb. The DGmb was mostly GFAP negative in all five animals. In general, the branches of the astrocytes in the hippocampus of three of the rats had short, thin branches and a stellate form resembling that observed in the sham-operated rats. The corresponding branches in the other two animals were long and thin. Furthermore, many astrocytes in these two animals had an atrophic appearance that included the lack of a distinct cell body and branches creating an irregular pattern (Figure 1G). 

*In The Aβ*_*1–40*_ *–vehicle* (*Cremophor EL*) *group* (*n = 4*), the DAPI-stained sections revealed signs of neuronal loss in the DGlb of all of the rats. The brains of these animals showed a pattern of gliosis that was very similar to that observed in the brains of the rats given only Aβ injection and hence will not be discussed further. Overall, astrocytes in the hippocampus of the four animals in this group had long branches of varying thickness, and many of them displayed the atrophic pattern described for the genistein-treated rats. 

### Quantitative observations

**Figure 2:**
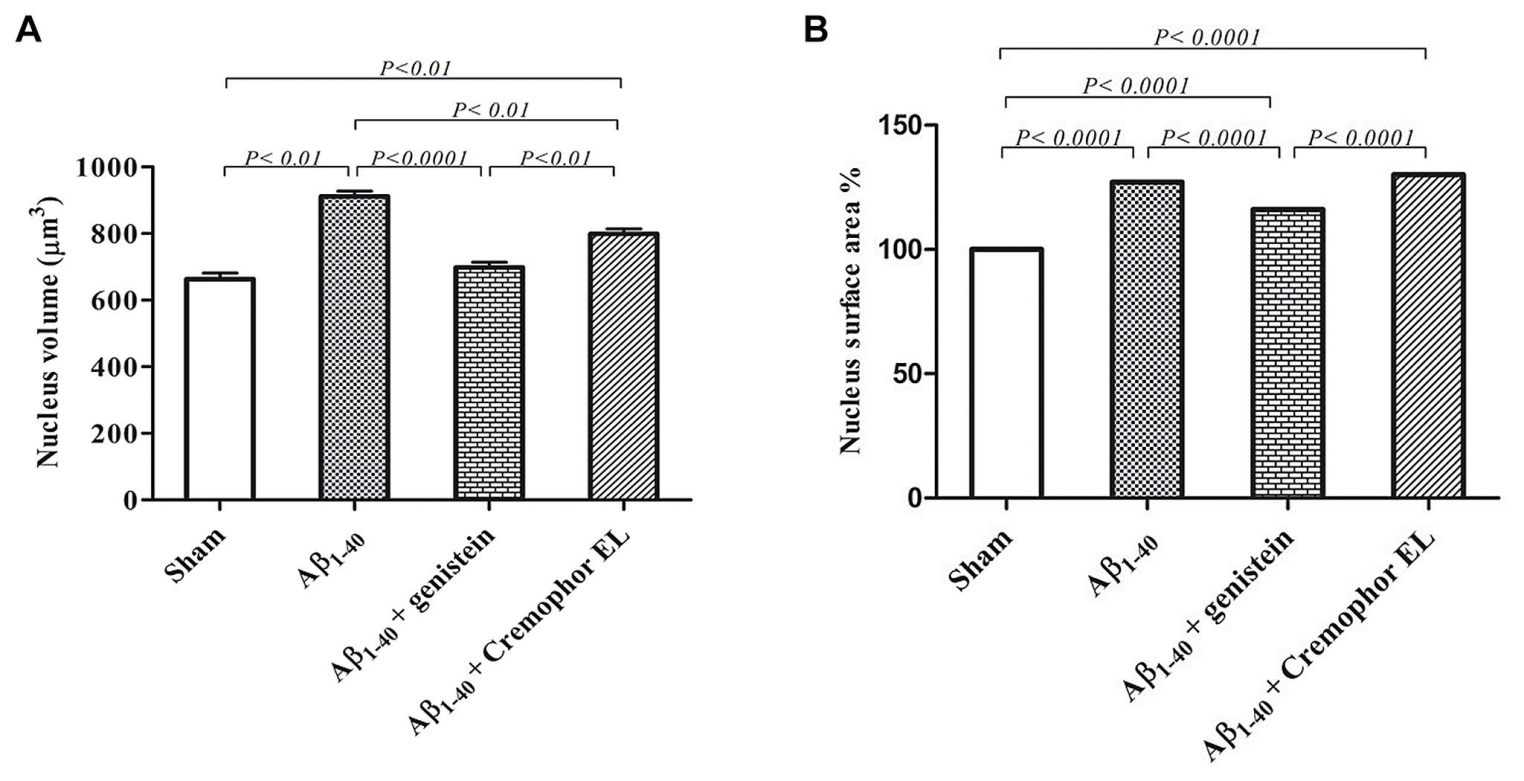
Compared to sham-operated rats, the mean volume (A) and surface area (B) of the astrocyte nucleus were increased in animals that received an Aβ injection in the hippocampus (n = 5), an Aβ injection plus genistein treatment (area only; n = 5), or an Aβ injection plus Cremophor EL treatment (n = 4). Cremophor EL was used as a vehicle for genistein. Values are means ± SEM. The nucleus of fifty astrocytes per group were evaluated. n = number of rats.

**Figure 3:**
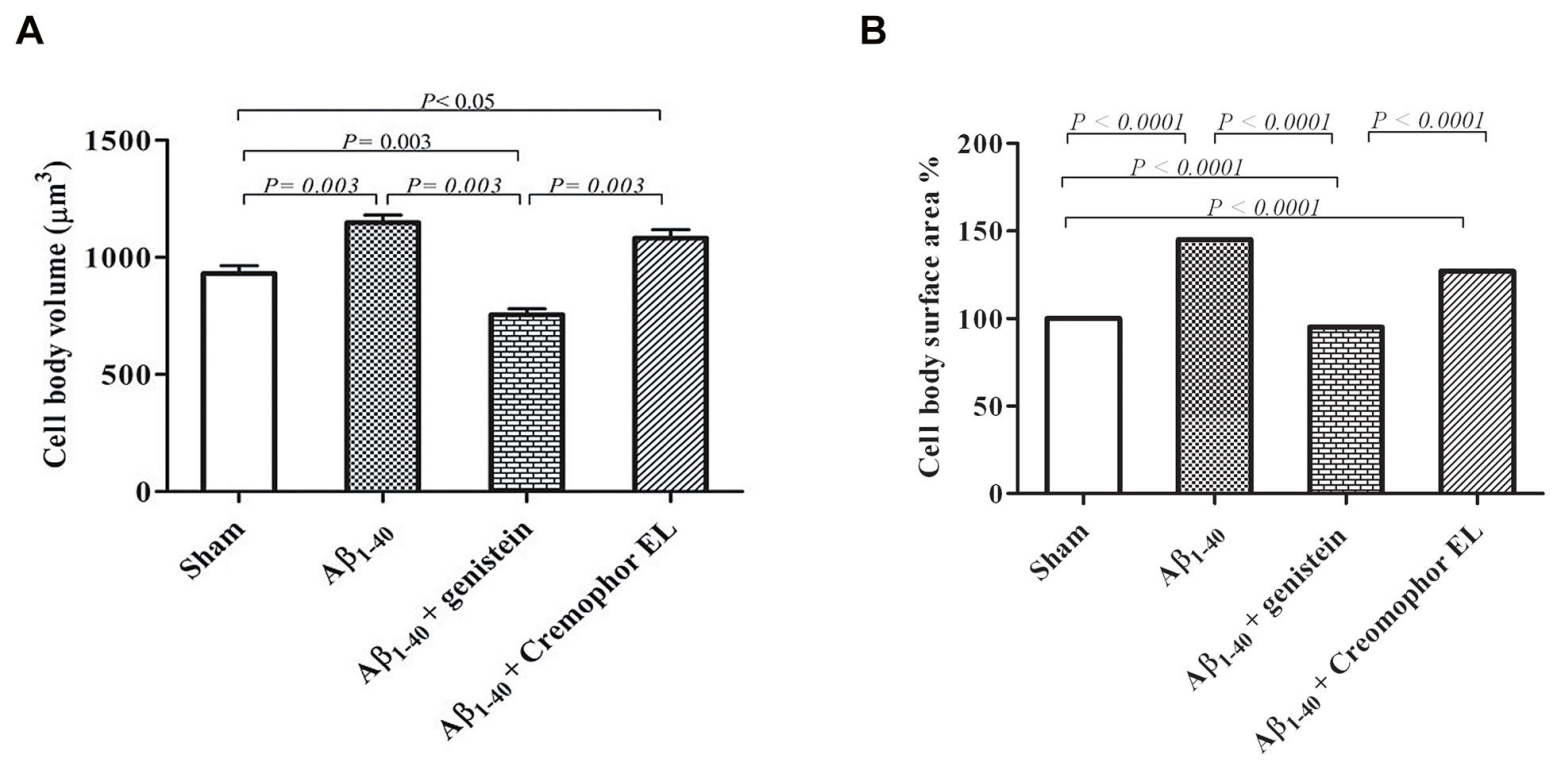
The mean volume (A) and surface area (B) of the cell body of astrocytes measured in rats subjected to sham operation (n = 4), Aβ_1–40_ injection (n = 5), Aβ_1–40_ injection plus genistein treatment (n = 5), or Aβ_1–40_ injection plus vehicle (Cremophor EL; n = 4) treatment. Genistein treatment inhibited the Aβ_1-40_-induced cell body enlargement (vs. Aβ_1-40_ injection) and also significantly ameliorated the enlargement caused by the insertion of the needle (vs. sham operation). Astrocyte cell body size was increased in the Cremophor EL injection group compared to the sham-operated and the Aβ_1–40_-injected–genistein-treated rats. Cremophor EL was used as a vehicle for genistein. Values are means ± SEM. Fifty astrocytes per group were evaluated. n = number of rats.

**Figure 4:**
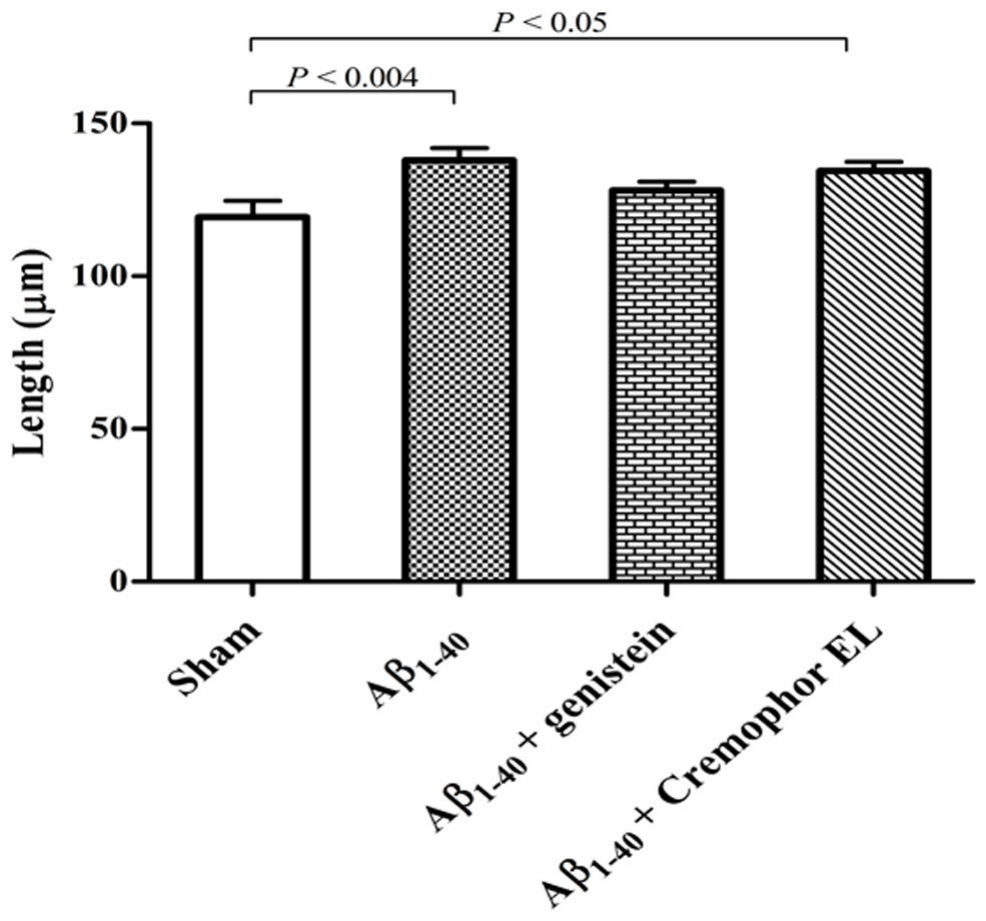
Compared to sham-operated rats (n = 4), the total length of astrocytic branches was increased in Aβ_1–40_-injected (n = 5) and Aβ_1–40_-Cremophor-EL-treated rats (n = 4), but not in Aβ_1–40_-genistein-treated rats (n = 5). Cremophor EL was used as a vehicle for genistein. Values are means ± SEM. Fifty astrocytes per group were included in the evaluation. n= number of rats.

**Figure 5:**
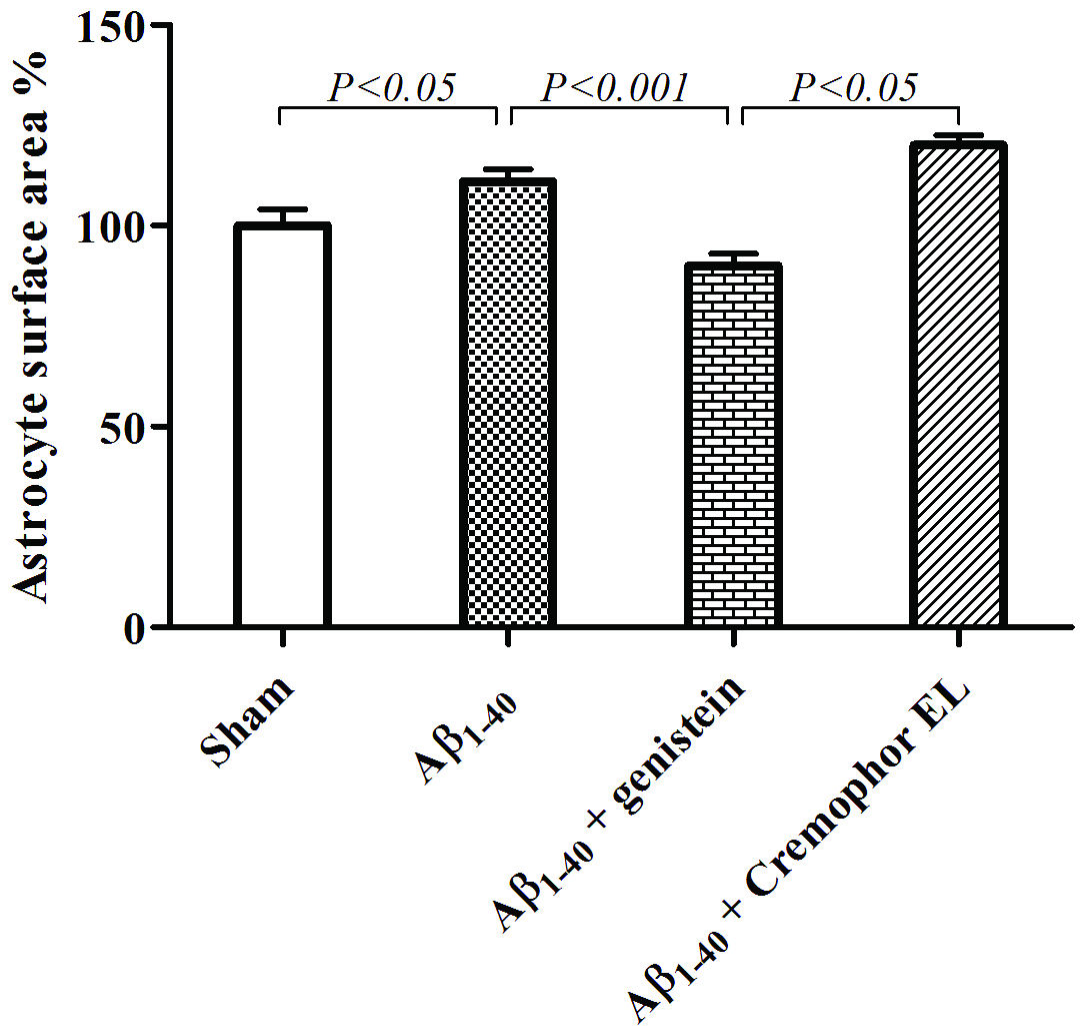
The mean volume (A) and surface area (B) of astrocytes (cell body + branches) was increased in Aβ_1–40_-injected rats (n = 5), and this enlargement was inhibited by genistein (n = 5) but not by Cremophor EL (n = 4). Cremophor EL was used as a vehicle for genistein. Values are means ± SEM. Fifty astrocytes per group were included in the evaluation. n = number of rats.

**Figure 6:**
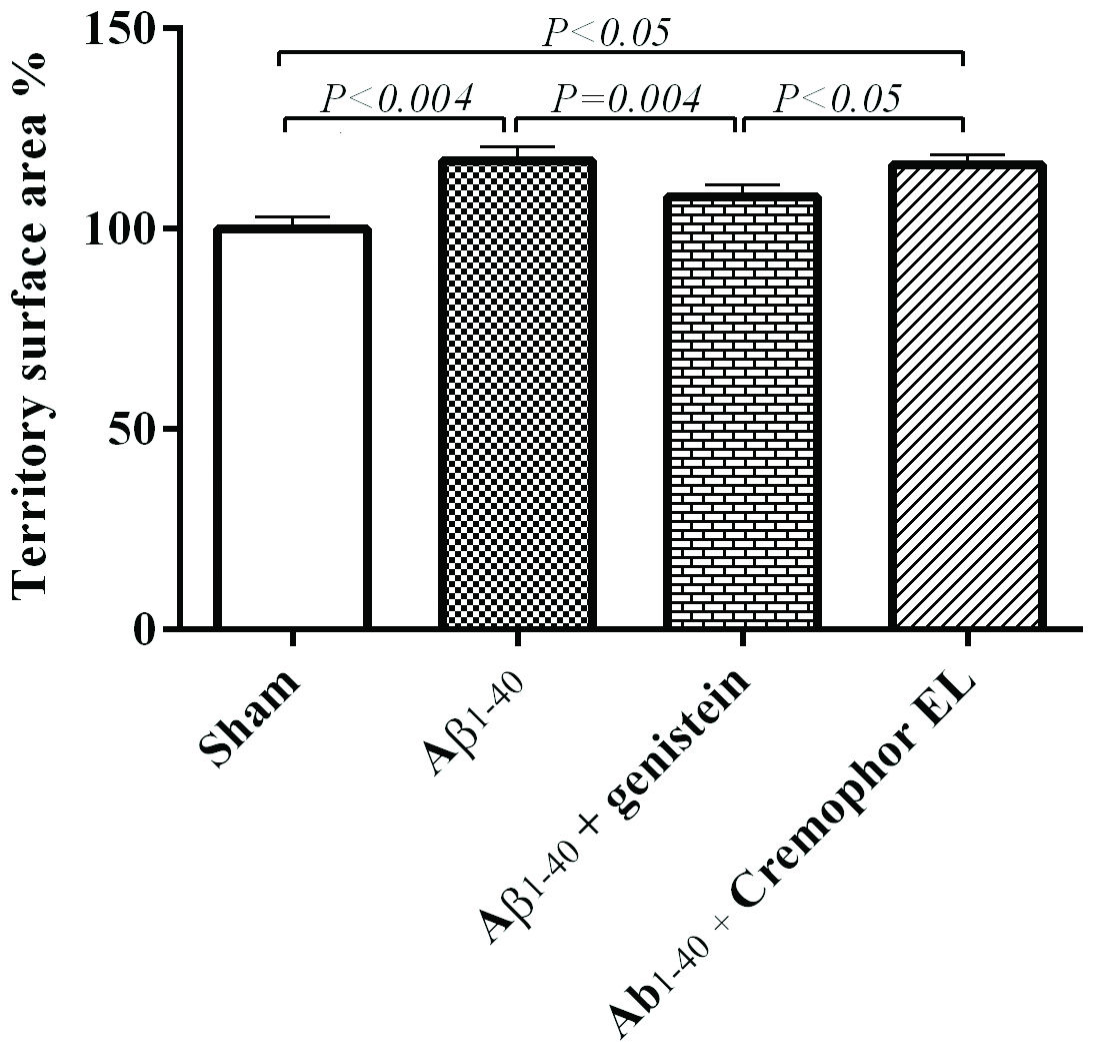
The mean volume (A) and surface area (B) of astrocyte tissue territory were increased in the Aβ_1–40_-injected (n = 5) and Aβ_1–40_-Cremophor-EL-treated groups (n = 4), but not in the Aβ_1–40_-genistein-treated rats (n = 5). Cremophor EL was used as a vehicle for genistein. Values are means ± SEM. Fifty astrocytes per group were included in the evaluation. n = number of rats.

**Figure 7:**
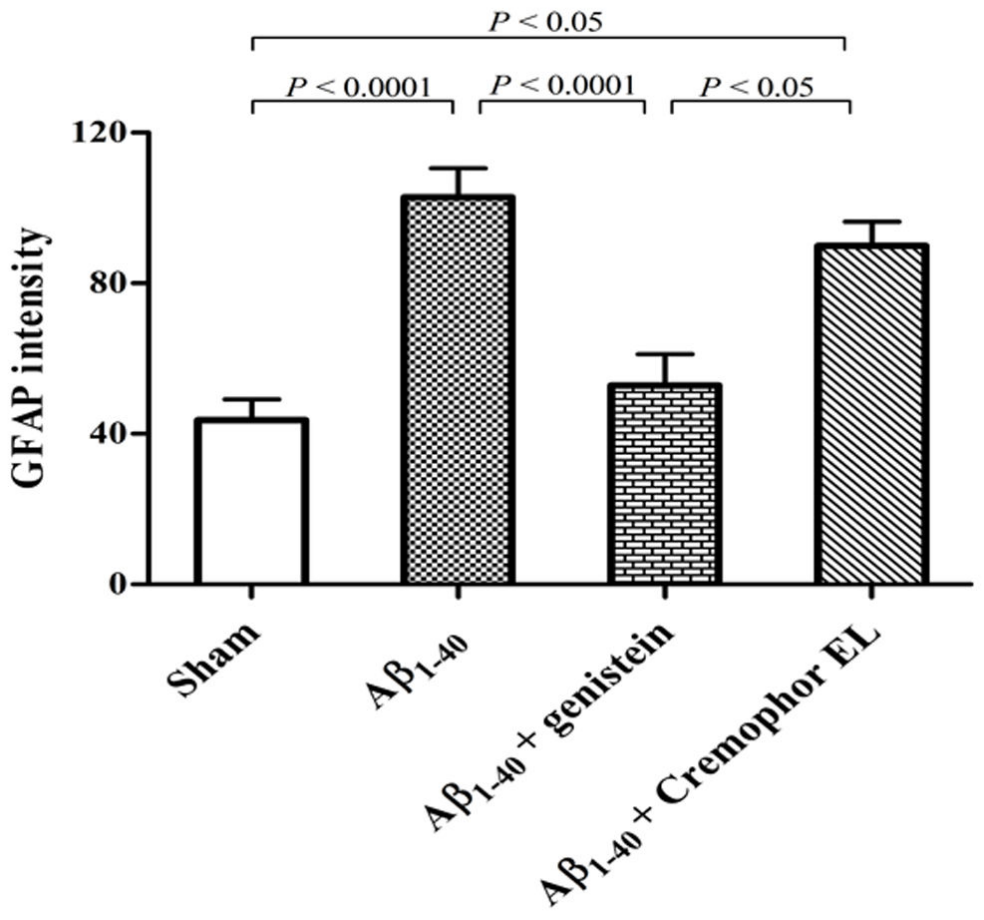
The intensity of GFAP^+^ immunoreactivity was increased in the Aβ_1–40_-injected (n = 5) and Aβ_1–40_-Cremophor-EL-treated rats (n = 4), but not in the Aβ_1–40_-genistein-treated animals (n = 5). Cremophor EL was used as a vehicle for genistein. Values are means ± SEM. For each animal two brain sections were evaluated. n = number of rats.

All values obtained for the Aβ_1–40_-injected–Cremophor-EL-treated rats (except measurements of the surface area of both the cell body and entire astrocytes) differed significantly from the corresponding values for the sham-operated rats but showed the same pattern as in the values for the Aβ_1–40_-injected rats. Therefore, data on the Aβ_1–40_-Cremophor-EL group are presented only in Table 1 and Figures 2–7 (i.e., are not given further consideration below).

**Table 1:** Morphometric analysis of GFAP^+^ astrocytes in rat hippocampus.

	**Sham–operated**	**Aβ–injected**	**Aβ–injected + genistein**	**Aβ–injected + CremophorEL**
	**n = 50**	**n = 50**	**n = 50**	**n = 50**
**Nucleus volume (µm^3^)**	**663 ± 18**	**911 ± 16**	**698 ± 16**	**799 ±15**
			**P < 0.0001	* *P* < 0.01
		* *P* < 0.01	δ*P* < 0.01	** *P* < 0.01
**Cell body volume (µm^3^)**	**930 ± 34**	**1148 ± 33**	**754 ± 26**	**1082 ± 36**
		* *P* = 0.003	* *P* = 0.003	* *P* < 0.05
			**P = 0.003	
			δ*P* = 0.003	
**Total length of branches (µm**	**119.4 ± 5.3**	**138.0 ± 4.0**	**128.1 ± 2.8**	**130.4 ± 2.9**
		* *P* = 0.004		* *P* < 0.05
**Astrocyte volume (µm^3^)**	**5280 ± 215**	**5875 ± 168**	**4629 ± 164**	**5645 ± 125**
		* *P* = 0.03	* *P* = 0.01	* *P* < 0.05
			**P < 0.0001	
			δ*P* < 0.05	
**Territory volume (µm^3^)**	**16994 ± 634**	**20672 ± 604**	**15887 ± 486**	**19869 ± 489**
		* *P* < 0.0001	**P < 0.05	* *P* < 0.05
			δ*P* < 0.0001	
**GFAP intensity**	**43.7 ± 5.4**	**102.8 ±7.7**	**52.9 ± 8.3**	**89.9 ± 6.5**
		* *P* = 0.0001	**P = 0.0001	* *P* < 0.05
			δ*P* < 0.05	

NaCl (sham-operated) or Aβ_1-40_ (2 nM) was injected in the hippocampus. Genistein (10 mg/ kg) and Cremophor EL (0.5 ml/ rat) were administered by gavage. * *vs*. sham operated group, ** *vs*. Aβ-injected group, δ *vs*. Aβ-injected Cremophor EL treated group. Astrocyte surface area and volume = surface area and volume of cell body + branches. Territory surface area and volume = the surface area and volume of the tissue covered by individual astrocytes. n = number of astrocytes.

#### Astrocyte nucleus

The mean volume of the nucleus in the sham-operated rats was 663 µm^3^. Injection of Aβ_1–40_ led to a 37% increase in this parameter (*P*< 0.01; Table 1, Figure 2A) and a 27% increase in the surface area (*P*< 0.0001; Figure 2B). Genistein treatment prevented the Aβ_1–40_-induced increasein the volume of the nucleus and significantly decreased the increment of the surface area (*P*< 0.0001 vs. Aβ-injected rats; Table 1, Figure 2A,B).

#### Astrocyte cell body

In the sham-operated rats, the mean volume of the cell body of astrocytes was 930 µm^3^.

Injection of Aβ_1–40_ increased the cell body volume by 23% (*P* = 0.003; Table 1, Figure 3A) and the surface area by 43% (*P*< 0.0001; Figure 3B). The Aβ_1–40_-induced enlargement was significantly blocked by treatment with genistein (volume *P* = 0.003 and surface area *P*< 0.0001 vs. Aβ-injected rats; Table 1, Figure 3A,B). Compared with the sham-operated animals, the genistein-treated rats had astrocytes with a 19% smaller mean cell body volume (*P* = 0.003) and a 6% smaller surface area (*P*< 0.0001; Table 1, Figure 3A,B).

#### Astrocyte branches

A mean astrocyte in the sham-operation group exhibited 8.9 ± 0.3 GFAP^+^ branches; 68.5% were primary branches. In these respects, Aβ_1–40_ injection did not affect the astrocytes but genistein treatment significantly increased the number of branches compared to sham or Aβ_1–40_-injection group (Table 2). The mean length of the astrocyte branches in the sham-operated rats was 119.4 µm. This parameter was significantly increased (15%; *P* = 0.004) in the astrocytes of Aβ_1–40_ injected rats, and this elongation was inhibited by genistein (Table 1, Figure 4).

**Table 2:** Mean number of GFAP^+^ branches/astrocyte.

**Branches**	**Sham–operated**	**Aβ–injected**	**Aβ–injected + genistein**
	**n = 197**	**n = 247**	**n = 198**
**Total number**	**8.9 ± 0.3**	**8.4 ± 0.2**	**10.1 ± 0.2**
			*P < 0.001
			**P < 0.0001
**Primary branches**	**6.1 ± 0.2**	**5.9 ± 0.1**	**6.8 ± 0.2**
**(% of total number)**	**(68.5)**	**(70.2)**	**(67.3)**
			*P = 0.001
			**P = 0.0001

NaCl (sham-operated) or Aβ_1-40_ (2 nM) was injected into the hippocampus. Genistein (10 mg/ kg) was administered by gavage. n = number of astrocytes. * *vs.* sham operated group, ** *vs.* Aβ-injected group.

#### Astrocyte size (cell body + branches)

The astrocytes in the sham-operated rats had a mean volume of 5280 µm^3^. An increase of 11% in both the volume (*P* = 0.03) and the surface area (*P* < 0.05) of the astrocytes were observed when measurements were performed on tissue from Aβ_1–40_ injected animals, and both these increases were inhibited by genistein (*P* < 0.0001 and *P* < 0.001, respectively; Table 1, Figures 5A,B).

#### Astrocyte territory

To assess what we called the functional astrocyte territory, we measured the surface area and the volume of the tissue covered by individual astrocytes. Compared to astrocytes in the sham-operated rats, those in the animals that received an injection of Aβ_1–40_ showed a 22% increase in the mean territory volume (*P*< 0.0001) and a 17% increase in the surface area (*P*< 0.004); genistein inhibited the effect of Aβ_1–40_ on the volume and also lessened the impact of the amyloid on the surface area (Table 1, Figures 6A,B).

#### Astrocyte density

The mean number of astrocytes/1000 µm^2^, was 5.6 ± 0.05 in the sham-operated rats, 11.7 ± 0.1 in the Aβ_1–40_-injected rats, and 6.7 ± 0.05 in the Aβ_1–40_-genistein-treated animals. The higher astrocyte density in Aβ_1–40_-injected rats was significant in comparison with data from other groups (*P* < 0.0001).

#### GFAP Intensity

The mean intensity of the GFAP^+^ immunoreactivity was 43.7 ± 5.4 in the sham-operated group, 102.8 ± 7.7 in the Aβ_1–40_-injected rats, and 52.9 ± 8.3 in the Aβ_1–40_-genistein-treated animals. The results clearly showed that injection of Aβ increased the presence of GFAP^+^ astrocytes in the hippocampus by 135% (*P* = 0.0001), and this rise in immunoreactivity was significantly inhibited by genistein (Table 1, Figure 7).

### Protein composition in the gliotic hippocampus of Aβ_1–40_-injected rats

**Figure 8:**
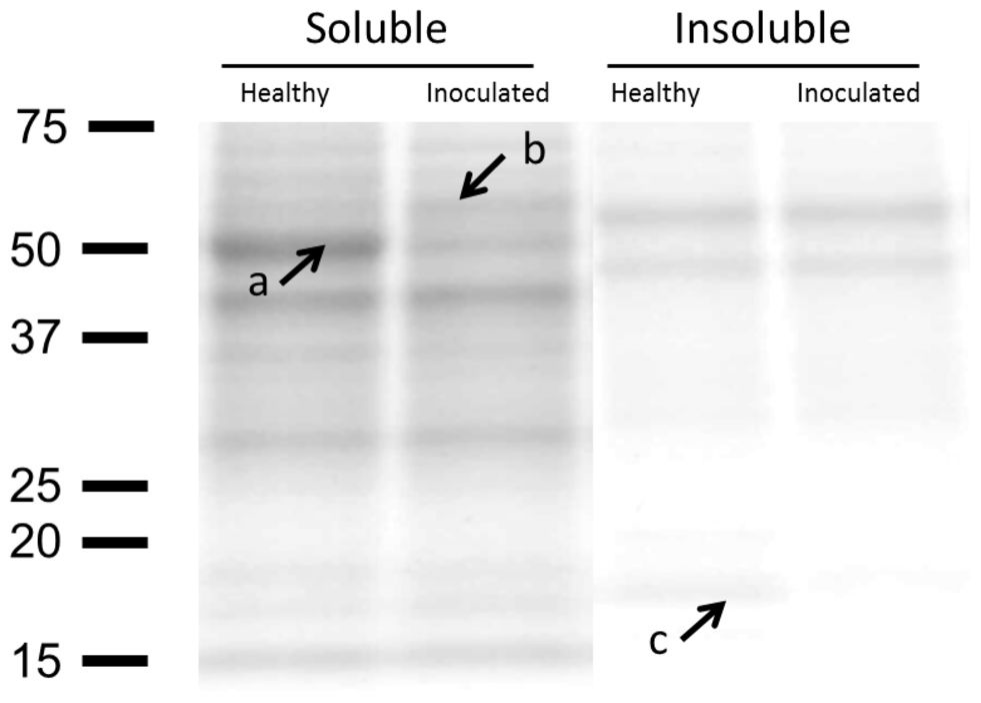
SDS-PAGE of hippocampal brain tissue homogenates for in-gel digestion and protein identification by mass spectrometry, comparing Aβ_1–40_ injected rats and healthy controls. Proteins identified as tublins and enolases (**a**) and myelin basic proteins (**c**) appear to decrease in Aβ_1–40_ injected rats indicative of neuronal loss. Proteins identified as dihydropyrimidinase-related protein 2 and pyruvate kinase M1/M2 (**b**), appear more abundant in Aβ_1–40_ injected rats compared to healthy control animals. Soluble and insoluble fractions of brain homogenate were isolated as described in Materials and Methods. The right and left hemispheres from three healthy and three Aβ_1–40_-injected rats were included.

SDS-PAGE with in gel digestion and mass spectrometric analysis revealed decrease of  tublin, enolase and myelin basic proteins, and increase of  dihydropyrimidinase-related protein 2 and pyruvate kinase M1/M2 in Aβ_1–40_-induced gliotic tissue in comparison to tissue taken from healthy animals (Figure 8; Table 3).

**Table 3:** Mass spectrometric analysis of brain hippocampus in Aβ_1–40_ injected rats and healthy animals.

**Band**	**Protein**		**Uniprot Accession number/Entry name**	**Theoretical MW, kDa**	**Number of peptides identified**	**Mascot protein score**
**A**	tubulin	beta-2B	Q3KRE8 (TBB2B_RAT)	50,361	16	**636**
		beta-3	Q4QRB4 (TBB3_RAT)	50,419	14	**582**
		beta-5	P69897 (TBB5_RAT)	49,671	14	**571**
		alpha-1A	P68370 (TBA1A_RAT)	50,894	15	**559**
	enolase	alpha	P04764 (ENOA_RAT)	47,440	11	**398**
		gamma	P07323 (ENOG_RAT)	47,510	4	**184**
**B**	dihydropyrimidinase-related protein 2		P47942 (DPYL2_RAT)	62,278	15	**683**
	pyruvate kinase M1/M2		P11980 (KPYM_RAT)	58,294	17	**534**
**C**	myelin basic protein		P02688 (MBP_RAT)	21,546	6	**149**

Three protein groups (**A**; tubulin and enolase, **B**; dihydropyrimidinase-related protein 2 and pyruvate kinase M1/M2, and **C**; myelin basic protein) were identified in rat hippocampus homogenate using in-gel digestion and LC-MS/MS analysis. Aβ_1-40_ injected rats showed less amount of proteins in group **A** and **C** and higher amount of proteins in group **B** in comparison with the homogenate of the brain tissue taken from healthy animals.

## Discussion

In the current study, we used antibodies against GFAP as a marker for identification of astrogliosis that is characterized by overexpression of GFAP, and pronounced hypertrophy of the cell body and their processes (9,10). We found that injection of Aβ_1–40_ into the rat brain was associated with astrocytic hypertrophy and this event was significantly inhibited by genistein. Previous studies have demonstrated that genistein has an anti-inflammatory impact on conditions such as diabetes [18] and on various types of tissue, including retina [19], gut [20], respiratory organs [21], kidney [22], and arthritic joints [23,24]. Genistein exerts its anti-inflammatory effect by influencing transcription factors, enzymes, and inflammatory mediators that are involved in inducing inflammation. For example, genistein decreases production of reactive oxygen species (ROS) [25], and it inhibits NF-κB and the signal transducer and activator of transcription 1 (STAT-1), which are transcription factors for nitric oxide synthase (iNOS) [26,27]. In addition, genistein prevents the hemolysate- or Aβ-mediated induction of iNOS, as well as other inflammatory mediators such as cyclooxygenase-2 (COX-2), prostaglandin E synthases (enzymes that are involved in the synthesis of prostaglandin E2), interleukin 1 beta (IL-1 beta), and tumor necrosis factor alpha (TNF-alpha) in primary cultures of astrocytes or macrophages [26,28,29]. Furthermore, it has been observed that intravitreal injection of genistein in diabetic rodents reduced inflammation and microglial activation in the retina by involving extracellular signal-regulated kinase (ERK) and P38 mitogen-activated protein kinases (MAPKs) in activated microglia [19]. Activation of p38 MAPK by genistein increases the export of iron from astrocytes via the estrogen receptor [30]. Considering activation of apoptotic pathways, it has been suggested that genistein induces apoptosis in glioblastoma cells, but not in normal human astrocytes, by eliciting production of ROS [31]. Thus, genistein can ameliorate inflammation through activation of a cascade of intracellular molecules.

### Changes in GFAP intensity

Compared with the sham-operated group, the Aβ_1–40_-injected rats in our study showed increased intensity of GFAP immunoreactivity in the hippocampus, but this was not found in the Aβ_1–40_-injected–genistein-treated animals. This increase can be explained partly by hypertrophy and partly by upregulation of the GFAP transcription in reactive astrocytes, which is considered to play a role in the events involved in gliosis [8,32-34]. In fact, the density of the GFAP^+^ astrocytes in the hippocampus of Aβ_1–40_-injected rats in the current study was raised over 200% compared to other groups. In conclusion, our results suggest that genistein can alleviate those cellular reactions that led to Aβ_1–40_-induced raise of GFAP intensity. 

### Changes in shape and size of astrocytes

Protoplasmic astrocytes are normally stellate in shape and have fine branches, although, depending on their location in the CNS, these cells can modify their own morphology and size [35]. This morphological transformation is a rapid process that requires redistribution of the cytoskeletal proteins [36]. For example, in a study of the rostral preoptic area of the hypothalamus [37], it was noted that the surface area of astrocytes that were in close apposition to neurons that produced gonadotropin-releasing hormone (GnRH) exhibited a decrease in surface area between the hours of 0800 and 1200, before the onset of the luteinizing hormone surge. In a diseased condition in the brain, such as that induced by the presence of a high level of Aβ peptide, astrocytic processes become convoluted and can exhibit swollen terminals [38]. In addition, both decreased and increased complexity and size of astrocytes have been observed in conditions such as hypoxia/ischemia [35,39]. The current results suggest that when Aβ_1–40_ is present in brain tissue, astrocytes become reactive. This assumption agrees with data reported by Garwood and colleagues [11] demonstrating the presence of hypertrophic astrocytes in proximity to senile plaques. The occurrence of reactive astrogliosis in tissue early after injury is considered to be beneficial, because it can reestablish the chemical environment by removing harmful molecules. Reactive astrocytes can also improve the physical environment by creating scar tissue to prevent harmful molecules from spreading to healthy parts of the tissue [40]. On the other hand, the scar tissue contains a dense network of astrocytes that release inhibitory molecules, which in turn reduce the ability of the tissue to recover [8]. Astrocyte activation is accompanied by elevated production of neurotoxic factors, including cytokines, NO, and ROS, which can induce neuronal death and brain atrophy [40]. Here, we found that rats that were treated only with Aβ_1–40_ exhibited more extensive astrogliosis than those that were given both Aβ_1–40_ and genistein. In one of our earlier studies [15], we noted that neuronal degeneration in hippocampus was also more severe in rats given only Aβ_1–40_ than in those that received both Aβ_1–40_ and genistein. In addition, the results of the current study showed significantly higher density of GFAP^+^ astrocytes in the Aβ_1–40_ exposed hippocampus.Together, these observations suggest that a positive correlation exists between the density of reactive astrogliosis and the severity of the damage in the tissue, which has also been proposed by other investigators [41-44].

As already mentioned, we found that injection of Aβ peptide into the hippocampus caused hypertrophy of the astrocytes in the damaged tissue. Hypertrophic astrocytes have been observed in patients with pathological conditions such as Alzheimer’s [45] and AIDS [46], and in depressed suicide subjects [47]. In studies thus far, morphometric analysis of astrogliosis has been performed on 2D photographs that only measure a fraction of a cell. Moreover, the investigations have varied considerably with regard to the choice of staining methods (i.e., histochemistry or immunohistochemistry) and techniques for creating micrographs, as well as the types of microscopes employed. For example, Hama and colleagues [48] measured the perimeter and area of the processes of the same astrocytes and obtained 2.06 times higher values when using high-voltage electron microscopy compared with light microscopy. The detection of GFAP is also limited, because this protein is not expressed in fine tertiary cell processes; according to Bushong and colleagues [1], this means that the method can only visualize 15% of an astrocyte. Consequently, it is not possible to compare measurements of astrocytes reported by different authors. Nonetheless, the question arises whether the sizes of astrocytes recorded in our investigation are reasonable. In our sham-operated rats, the brain was exposed to a needle that was inserted to inject NaCl in the tissue, and hence the astrocytes in these animals were probably hypertrophic due to the mechanical damage of the brain. To our knowledge, there are no reports describing measurement of the size of astrocytes in an animal model of AD similar to the one we used in the current study, and therefore it is not possible to compare our measurements with values published by other researchers. However, some data on astrocytic hypertrophy have been obtained in other animal models. Chvatal and colleagues [49] performed confocal microscopy on slices of cortex from transgenic GFAP/EGFP mice to evaluate the 3D size of astrocytes, and the results showed that the cell body volume was 14.6% of the total cell volume. Similarly, we found that the average cell body volume of astrocytes in our study was 17.6% of the total cell volume detected by GFAP. It should be mentioned that our rats were perfused with 4% PFA, whereas the mice studied by Chvatal and colleagues [49] were decapitated and each brain was placed in artificial cerebrospinal fluid before analysis. Regarding the hypertrophy, Anderova and colleagues [39] observed that the total astrocyte volume (cell body and branches) increased by 250% in rats one month after hypoxia/ischemia, and Girardi et al. [50] found that the astrocyte area increased by 300% in a rat model of epilepsy. By comparison, our data indicated an 11% increase in astrocyte area and volume three weeks after injection of Aβ_1–40_, a level that is quite low compared to the values reported in the cited investigations. However, it should be taken into consideration that astrocytes in our sham-operated group were already hypertrophic as a result of mechanical injury caused by insertion of a needle, and thus the increase we recorded represents additional hypertrophy initiated by the presence of the amyloid. This mechanical injury can partly contributed to the high density of GFAP^+^ astrocytes in the tissue of rats in our study.

### Protein composition in gliotic tissue

We analyzed protein composition of Aβ_1–40_-injected hippocampal tissue to validate the occurrence of Aβ-induced neuronal cell damage in the brain. The results of mass spectrometric analysis in the current study showed a weak presence of tubulin, enolase and myelin basic proteins in Aβ_1–40_-injected tissue compared with healthy tissue which signals the loss of neurons in the tissue as we have previously reported (15). In addition, there appeared to be an increased amount of the tubulin binding and axonal transport protein dihydropyrimidinase-related protein 2. This is well in line with proteomic analyses in transgenic AβPP mice [51]. The results of the current study is in agreement with our previous studies showing that intrahippocampal injection of the Aβ_1–40_ in rats caused extensive neuronal degeneration in the tissue [15] leading to impaired memory [14]. Furthermore, the study presented here indicated the presence of a higher level of pyruvate kinase M1/M2 in Aβ_1–40_-injected tissue indicating a high metabolic activity in the gliotic tissue. Since the gliotic tissue was deprived of neurons as discussed above, this high metabolic activity is likely related to glial cells.

### Method considerations

#### Regarding the genistein

genistein asserts its beneficial effect by its affinity to the estrogen receptor, stimulating the expression of antioxidants in normal condition, and inhibition of DNA synthesis in cancer cells as discussed above. A high dietary level of this compound, however, can have inhibitory effect on tyrosine kinase [52] and therefore, can impair long-term potentiation (LTP) in the hippocampus. Furthermore, Kim et al. [53] showed that high concentration of genistein can have toxic effects on the development of zebrafish embryos. In the current study, we used a single dose of 10 mg/kg genistein since the daily consumption of this amount was shown to improve the values of biomarkers in clinical studies of Sanfilippo syndrome Patients without significant side effects [54,55]. Furthermore, Morán and colleagues [56] did not find any beneficial effect of a higher concentration (40 mg/kg) on homeostasis in rat cerebral cortex in comparison to a dose of 10 mg/kg. 

#### Regarding the time window for our observation

in the current study, we performed our observation three weeks after hippocampal Aβ_1–40_ injection. We chose this time window since in our previous studies we did not observe any alteration in learning and memory in rats before day 14 after Aβ_1–40_ injection. At day 14 to 20 post-surgery, however, a significant behavioral, biochemical and morphological alteration was found in the rats (14,15). The aim of the current study was to evaluate the morphological response of astrocytes to the presence of Aβ_1–40_ in the rat brain before and after treatment with genistein, and for that reason, we studied astrocytes when the cell damage should be significant and the presence of astrogliosis would be expected in the tissue i.e. 3 weeks after Aβ_1–40_ injection. The limitation of our research design is that the long-term development of the astrogliosis remains unknown.

## Concluding Remarks

In conclusion, we used 3D confocal microscopy to quantify morphological changes of reactive astrocytes and found that presence of Aβ_1–40_ in the tissue caused astrogliosis. Proteomic assessment indicated neuronal loss and enhanced metabolic activity of astrocytes responding to damage caused by Aβ_1–40_. Our findings also demonstrate that genistein can significantly ameliorate Aβ_1–40_-induced astrogliosis.
